# Silencing of long non-coding RNA H19 downregulates CTCF to protect against atherosclerosis by upregulating PKD1 expression in ApoE knockout mice

**DOI:** 10.18632/aging.102388

**Published:** 2019-11-22

**Authors:** Yongyao Yang, Feng Tang, Fang Wei, Long Yang, Chunyan Kuang, Hongming Zhang, Jiusheng Deng, Qiang Wu

**Affiliations:** 1 Department of Cardiology, Guizhou Provincial People’s Hospital, Guiyang 550002, P. R. China; 2 Department of Cardiology, The General Hospital of Ji’nan Military Region, Ji’nan 250031, P. R. China; 3 Department of Pathology and Laboratory Medicine, Emory University School of Medicine, Atlanta, GA 30322, USA

**Keywords:** long non-coding RNA H19, atherosclerosis, CCCTC-binding factor, polycystic kidney disease 1, atherosclerotic vulnerable plaque formation

## Abstract

This study aimed to explore the interactions among long non-coding RNA H19, transcriptional factor CCCTC-binding factor (CTCF) and polycystic kidney disease 1 (PKD1), and to investigate its potentially regulatory effect on vulnerable plaque formation and angiogenesis of atherosclerosis. We established an atherosclerosis mouse model in ApoE knockout mice, followed by gain- and loss-of-function approaches. H19 was upregulated in aortic tissues of atherosclerosis mice, but silencing of H19 significantly inhibited atherosclerotic vulnerable plaque formation and intraplaque angiogenesis, accompanied by a downregulated expression of MMP-2, VEGF, and p53 and an upregulated expression of TIMP-1. Moreover, opposite results were found in the aortic tissues of atherosclerosis mice treated with H19 or CTCF overexpression. H19 was capable of recruiting CTCF to suppress PKD1, thus promoting atherosclerotic vulnerable plaque formation and intraplaque angiogenesis in atherosclerosis mice. The present study provides evidence that H19 recruits CTCF to downregulate the expression of PKD1, thereby promoting vulnerable plaque formation and intraplaque angiogenesis in mice with atherosclerosis.

## INTRODUCTION

Atherosclerosis (AS), as the leading cause of stroke and myocardial infarction is prone to result in disability and mortality accounting for more than 50% of casualty cases worldwide [1]. The involvement of chronic inflammation is acknowledged to be an intrinsic factor in the initiation and progression of AS, ultimately leading to plaque rupture, thrombosis and vascular occlusion [2]. Vulnerable plaques, also known as unstable plaques, are mainly characterized by inflammatory infiltration, intraplaque hemorrhage, and angiogenesis [3]. Moreover, the rupture of unstable atherosclerotic plaques is a precursor of acute coronary syndromes, known as a major complication of AS. Pathological angiogenesis of the vessel is a frequently described characteristic aspect of the development and progression of atherosclerotic plaques in AS [4]. Angiogenesis plays an important role in both the initiation and late plaque rupture that lead to stroke and myocardial infarction [5]. Due to limitations of the available therapeutic pathways that might effectively reduce atherosclerotic vulnerable plaque formation and intraplaque angiogenesis, there is an urgent need for developing novel approaches to treat AS. Previous reports have flagged the involvement of long non-coding RNA (lncRNA) in the regulation of AS [6], which provides new insight for a preclinical investigation based on lncRNA-targeted therapy.

LncRNAs are demonstrated to impact upon AS progression through their regulatory roles in the metabolism of endothelial cells and smooth muscle cells. For example, upregulated lncRNA H19 has been documented in the blood of patients with coronary artery disease [7]. Importantly, H19 is also expressed at a high level in blood examinations conducted for patients with AS and furthermore, human umbilical vein endothelial cells overexpressing H19 have exhibited improved proliferation and suppressed apoptosis [8]. An earlier study drew attention to the contribution of H19 knockdown to impeded proliferation and promoted apoptosis of oxidized low-density lipoprotein (ox-LDL) stimulated human aorta vascular smooth muscle cells (VSMCs) via regulation of the miR-148b/WNT/β-catenin axis [9]. Reports have documented a high expression H19 in patients with AS, where H19 inhibits vascular endothelial cell apoptosis in arteriosclerosis obliterans through regulation of the mitogen-activated protein kinase/nuclear factor kappa-B signaling pathway [8, 10]. Meanwhile, H19 has been elucidated to upregulate acid phosphatase 5 protein levels, thereby promoting AS and amplifying the risk of ischemic stroke [11]. Additionally, it has been noted that up-regulation of H19 in the whole blood of patients with coronary artery disease serves as a predictive marker for AS progression [7]. CCCTC-binding factor (CTCF) is a versatile transcriptional regulator with diverse functionality in the genome such as transcriptional activation or repression [12]. CTCF has been identified as an essential factor for embryogenesis due to its involvement in promoting mouse vascular development [13]. CTCF has been found to cooperate with lncRNA HOXA transcript at the distal tip (HOTTIP) to further regulate the expression of HOXA [14].

Polycystic kidney disease 1 (PKD1) and PKD2 are genes responsible for encoding polycystin 1 and polycystin 2 respectively, and loss-of-function of PKD1 and PKD2 genes can result in autosomal dominant polycystic kidney disease in subsequent generations [15]. Polycystins is demonstrated to inhibit renal epithelial cell apoptosis provoked by mechanical stress through mediating the opening of stretch-activated K2P channels, in which is the site for undergoing pressure or flow stimulation alterations such as in heart failure or atherosclerosis [16]. PKD1 has been identified as a novel risk gene for coronary artery disease in patients suffering from type 1 diabetes, according to a genome-wide association study [17]. Besides, PKD1 has been reported to be involved in AS in association with activation of p38 and p53, implying that interactions between these factors may be significant for the induction of AS [18]. Therefore, we hypothesized that H19 might promote AS by downregulating PKD1 through effects on CTCF, which we tested by inducing or silencing H19 in an AS model in established apolipoprotein E (ApoE) knockout mice.

## RESULTS

### H19 is highly expressed in aortic tissues of ApoE knockout mice with AS

**Figure 1:**
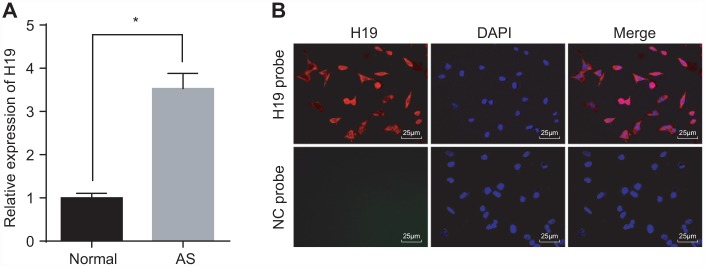
**High expression of H19 is found in AS aortic tissues.** (**A**) The expression pattern of H19 in normal and AS aortic tissues determined by RT-qPCR. * *p* < 0.05 *vs.* the control group. The data were measurement data and expressed by mean ± standard deviation. Data differences between two groups were analyzed by unpaired *t*-test. n = 6. (**B**) The subcellular localization of H19 detected by FISH assay (× 400). The experiment was repeated three times independently.

Initially, we conducted RT-qPCR to investigate the expression of H19 in the aortic tissues of normal and AS mice. The results indicated a higher level of H19 in the AS group compared to the control group (*p* < 0.05) (Figure 1A). Then, FISH assay was employed to explore the subcellular localization of H19, which proved to be present at high concentrations both in nuclei and cytoplasm (Figure 1B). Critical evidence based on examination of human atherosclerosis specimens suggested that H19 was predominantly expressed in the endothelial cell, where its expression was significantly down-regulated in pathological samples compared with healthy carotid artery biopsies [19]. These findings illustrated that elevated H19 in AS aortic tissues might potentially be involved in the pathophysiological process of AS.

### Silencing of H19 inhibits vulnerable plaque formation and intraplaque angiogenesis of ApoE knockout mice with AS

**Figure 2:**
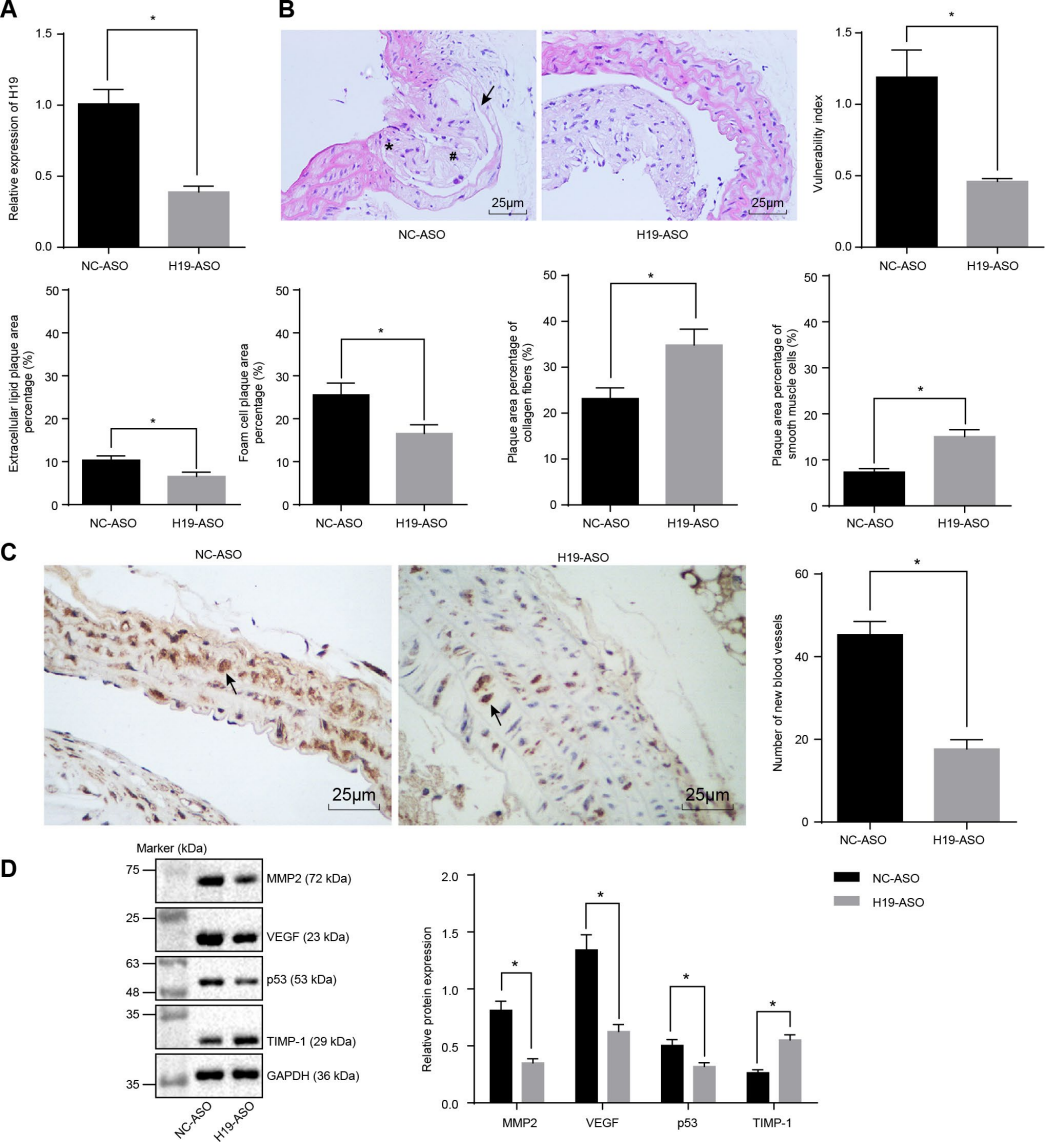
**Atherosclerotic vulnerable plaque formation and intraplaque angiogenesis of ApoE knockout mice with AS are inhibited by H19 silencing.** (**A**) The silencing efficiency of H19 assessed by RT-qPCR. * *p* < 0.05 *vs.* the NC-ASO group. (**B**) The atherosclerotic vulnerable plaque formation evaluated by HE staining (× 400) (The arrow referred to lipid vacuoles, * represented inflammatory cells and # indicated fractured smooth muscle.). (**C**) The number of new blood vessels measured by Immunohistochemical staining (× 400) (The arrow referred to CD34-positive cells). (**D**) The protein levels of MMP-2, VEGF, p53 and TIMP-1 in atherosclerotic plaques normalized to GAPDH after H19 silencing determined by Western blot analysis. * *p* < 0.05 *vs.* the NC-ASO group. The data were measurement data and expressed by mean ± standard deviation. Data differences between two groups were analyzed by unpaired *t*-test. n = 6. The experiment was repeated three times independently.

We next conducted RT-qPCR to assess the silencing efficiency of H19 in ApoE knockout mice with AS. In comparison with the NC-ASO group, the level of H19 was significantly lower in aortic tissues of the H19-ASO group (*p* < 0.05) (Figure 2A). Next, the intimal wall thickening, plaque formation, and plaque vulnerability index scores were evaluated after performing HE staining in order to examine the vulnerable plaque formation following H19 silencing. The NC-ASO group displayed a larger area of atherosclerotic plaques and thinner fibrous caps in addition to enlarged lipid plaque cores. Moreover, a large number of foam cells and deposited cholesterol crystals were observable within the atherosclerotic plaques. Additionally, the inner wall of the artery was thickened however the muscle layer was weakened. The observed atherosclerotic plaque was in an unstable state. A large amount of lipid vacuoles and macrophage infiltration were evident. The smooth muscle layer was thin, with a lack in type I and III collagen fibers. As for the H19-ASO group, we saw a smaller area of atherosclerotic plaques, smooth arterial inner walls and more fibrous caps without sign of fracture. Moreover, no distinct fracture and hemorrhage was evident within the atherosclerotic plaques of these mice. Additionally, a higher quantity of smooth muscle cells and a larger content of type I and III collagen fibers were observed. Moreover, a large number of foam cells accumulated in the atherosclerotic plaques. The cholesterol crystals were asymmetrically distributed, with calcification in some crystals. In general, the atherosclerotic plaques appeared to be in a stable state. The atherosclerotic plaques were less vulnerable, with a lower plaque vulnerability index in the H19-ASO group compared to the NC-ASO group (*p* < 0.05) (Figure 2B). These results provided ample evidence supporting that silencing of H19 could suppress atherosclerotic vulnerable plaque formation in ApoE knockout mice with AS.

To investigate the effect of H19 silencing on intraplaque angiogenesis of ApoE knockout mice with AS, immunohistochemical staining was performed to observe the angiogenesis, the arrow referring to CD34-positive cells. The results illustrated the expression of CD34 in vascular endothelial cells, raising the possibility of their involvement in intraplaque angiogenesis. A large number of newly formed blood vessels were observed in the NC-ASO group, which was significantly lower in the H19-ASO group (*p* < 0.05) (Figure 2C). Furthermore, Western blot analysis showed significantly decreased protein levels of MMP-2, VEGF and p53 along with an increased protein level of TIMP-1 in the H19-ASO group compared to the NC-ASO group (*p* < 0.05) (Figure 2D). These obtained results emphasized that H19 silencing repressed intraplaque angiogenesis of ApoE knockout mice with AS.

### Silencing of H19 upregulates the level of PKD1

**Figure 3:**
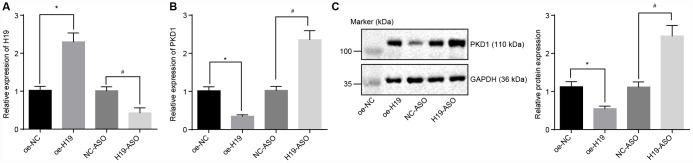
**The level of PKD1 is elevated by silencing of H19.** (**A**) The silencing or overexpressing efficiency of H19 assessed by RT-qPCR. (**B**) The transcriptional level of PKD1 after silencing or overexpressing H19 in atherosclerotic tissues determined by RT-qPCR. (**C**) The protein level of PKD1 after silencing or overexpressing H19 in atherosclerotic tissues normalized to GAPDH evaluated by Western blot analysis (the unprocessed blots are shown in Supplementary Figure 1). * *p* < 0.05 *vs.* the oe-NC group; # *p* < 0.05 *vs.* the NC-ASO group. The data were measurement data and expressed by mean ± standard deviation. Data differences between two groups were analyzed by unpaired *t*-test. n = 6. The experiment was repeated three times independently.

Initially, H19 was overexpressed or knocked down in the atherosclerotic tissues and was evaluated by RT-qPCR. In comparison with the oe-NC group, the expression of H19 was distinctly upregulated in the oe-H19 group, but notably downregulated in the H19-ASO group compared to the NC-ASO group (all *p* < 0.05) (Figure 3A), thus confirming the successful transfection of oe-H19 and H19-ASO into AS aortic tissues.. RT-qPCR and Western blot analysis showed that in comparison with the oe-NC group, the mRNA and protein level of PKD1 was significantly lower in the oe-H19 group, but markedly elevated in the H19-ASO group compared to the NC-ASO group (all *p* < 0.05) (Figure 3B, 3C), highlighting that H19 silencing increased the transcriptional as well as the protein level of PKD1 in aortic tissues of AS mice.

### H19 inhibits the transcriptional level of PKD1 via binding to CTCF transcription factor

**Figure 4:**
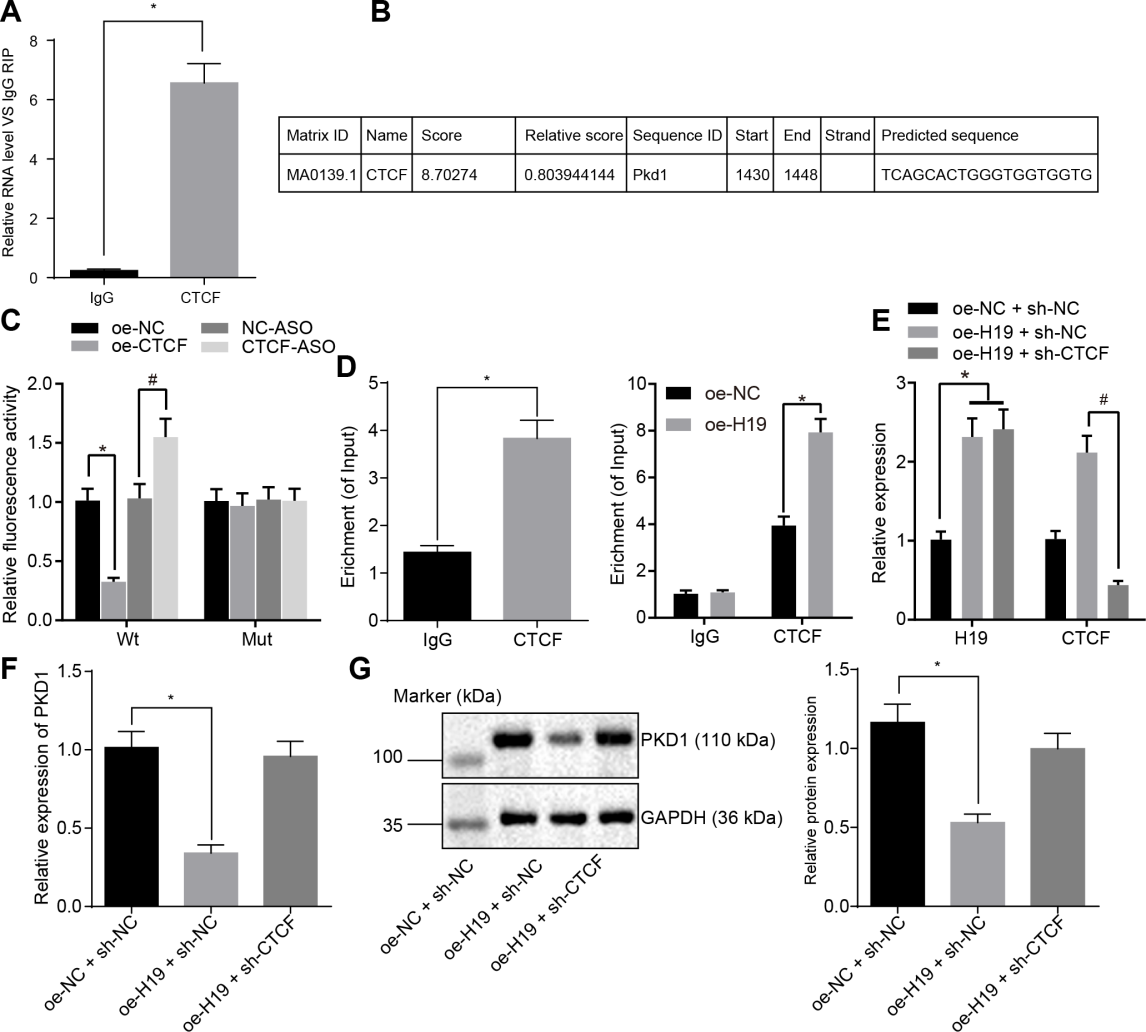
**H19 is capable of suppressing the transcriptional level of PKD1 through binding to CTCF.** (**A**) The binding ability of H19 to CTCF assessed by RIP. *, *p* < 0.05 *vs*. IgG. (**B**) The predicted binding site of CTCF on the promoter region of PKD1 through JASPAR (http://jaspar.genereg.net/). (**C**) The luciferase activity of PKD1-Wt/Mut after transfection of oe-CTCF or CTCF-ASO determined by dual-luciferase reporter gene assay. * *p* < 0.05 *vs*. the oe-NC group; # *p* < 0.05 *vs*. the NC-ASO group. (**D**) The binding ability of CTCF to the promoter region of PKD1 evaluated by ChIP assay. * *p* < 0.05 *vs*. IgG. (**E**) The transfection efficiency of oe-H19 and sh-CTCF determined by RT-qPCR. * *p* < 0.05 *vs*. the oe-H19 + sh-NC group. (**F**, **G**) The mRNA and protein level of PKD1 normalized to GAPDH after overexpressing H19 or knocking down CTCF measured by RT-qPCR and Western blot analysis (the unprocessed blots are shown in Supplementary Figure 2). * *p* < 0.05 *vs*. the oe-H19 + sh-NC group. The data were measurement data and expressed by mean ± standard deviation. Data differences between two groups were analyzed by unpaired *t*-test; comparisons made among multiple groups were analyzed by one-way ANOVA. n = 6. The experiments were repeated three times independently.

To evaluate the regulatory mechanism of H19 on the transcriptional level of PKD1 and to test whether CTCF was involved in the regulation of PKD1 by H19, we used a RIP assay to assess the binding ability of H19 to CTCF. In comparison with the IgG group, H19 enrichment was significantly upregulated in the CTCF group (*p* < 0.05) (Figure 4A), suggesting that H19 could potentially bind to CTCF. We next identified the potential binding sites of CTCF on the promoter region of PKD1 using JASPAR (http://jaspar.genereg.net/) (Figure 4B). To further verify the binding relationship between CTCF and PKD1, we adopted the dual-luciferase reporter gene assay. In comparison with the oe-NC group, the luciferase activity of PKD1-Wt was decreased in the oe-CTCF group. However, the luciferase activity of PKD1-Wt was higher in the CTCF-ASO group in comparison with the NC-ASO group (all *p* < 0.05). There were no significant differences in luciferase activity of PKD1-Mut compared to the different groups (*p* > 0.05) (Figure 4C). ChIP assay was adopted to further assess the binding ability of CTCF to the promoter region of PKD1, which showed the CTCF enrichment in the promoter region of PKD1 was significantly amplified in the CTCF group in comparison with the IgG group (*p* < 0.05) (Figure 4D), suggesting that CTCF could successfully bind to the promoter region of PKD1 and that overexpressed H19 induced more binding between CTCF and PKD1.

Next, we evaluated the level of PKD1 in cells overexpressing H19 or with knock down of CTCF. RT-qPCR examination of the transfection efficiency of oe-H19 and sh-CTCF (Figure 4E) showed that in comparison with the oe-NC + sh-NC group, the expression of H19 was upregulated in the oe-H19 + sh-NC group and the oe-H19 + sh-CTCF group (*p* < 0.05). When compared with the oe-H19 + sh-NC group, level of CTCF was downregulated in the oe-H19 + sh-CTCF group (*p* < 0.05), suggesting that oe-H19 and sh-CTCF were effective. The mRNA and protein level of PKD1 detected by RT-qPCR and Western blot analysis (Figure 4F, 4G) showed that the mRNA and protein level of PKD1 was notably lower in the oe-H19 + sh-NC group compared to the oe-NC + sh-NC group (*p* < 0.05), but it was salvaged in the oe-H19 + sh-CTCF group (*p* > 0.05). Therefore, we concluded that H19 suppresses the transcriptional level of PKD1 by binding to CTCF. When H19 was overexpressed, the level of CTCF was unregulated, which would bind to PKD1 and suppress the transcriptional level of PKD1.

### H19 participates in atherosclerotic vulnerable plaque formation and intraplaque angiogenesis by down-regulating PKD1 through recruiting CTCF in ApoE knockout mice with AS

**Figure 5:**
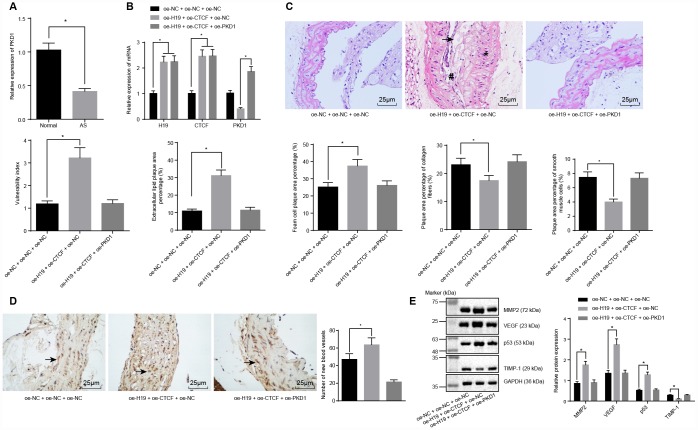
**H19 is involved in atherosclerotic vulnerable plaque formation and intraplaque angiogenesis through down-regulating PKD1 by recruiting CTCF in ApoE knockout mice with AS.** (**A**) The expression pattern of PKD1 in the aortic tissues of normal and AS mice determined by RT-qPCR. * *p* < 0.05 *vs.* the control group. (**B**) The overexpressing efficiency of H19, CTCF and PKD1 assessed by RT-qPCR. * *p* < 0.05 *vs.* the oe-NC + oe-NC + oe-NC group; # *p* < 0.05 *vs.* the oe-H19 + oe-CTCF + oe-NC group. (**C**) The atherosclerotic vulnerable plaque formation evaluated by HE staining (× 400) (The arrow referred to lipid vacuoles, * represented inflammatory cells and # indicated fractured smooth muscle). (**D**) The number of new blood vessels measured by Immunohistochemical staining (× 400) (The arrow referred to CD34-positive cells). (**E**) The protein levels of MMP-2, VEGF, p53 and TIMP-1 in atherosclerotic plaques normalized to GAPDH after transfection determined by Western blot analysis. * *p* < 0.05 *vs.* the oe-NC + oe-NC + oe-NC group. The data were measurement data and expressed by mean ± standard deviation. Data differences between two groups were analyzed by unpaired *t*-test; comparisons made among multiple groups were analyzed by one-way ANOVA. The experiments were repeated three times independently.

Previously, our results demonstrated that silencing of H19 could repress atherosclerotic vulnerable plaque formation and intraplaque angiogenesis of ApoE knockout mice with AS, however the underlying mechanisms remained unknown. Since H19 could inhibit the transcriptional level of PKD1 via binding to CTCF, we proposed a study to investigate whether PKD1 and CTCF were involved in the regulation of vulnerable plaque formation and intraplaque angiogenesis by H19. Initial RT-qPCR results showed that the level of PKD1 was downregulated in the AS group compared to the control group (*p* < 0.05) (Figure 5A). Subsequently, the ApoE knockout mice with AS were injected with plasmids overexpressing H19, CTCF or PKD1. In comparison with the oe-NC + oe-NC + oe-NC group, the levels of H19 and CTCF were significantly higher in AS aortic tissues of the oe-H19 + oe-CTCF + oe-NC group and the oe-H19 + oe-CTCF + oe-PKD1 group (*p* < 0.05). In comparison with the oe-H19 + oe-CTCF + oe-NC group, the level of PKD1 was notably elevated in the oe-H19 + oe- CTCF + oe-PKD1 group (*p* < 0.05) (Figure 5B).

HE staining and microscopic examination showed that, in comparison with the oe-H19 + oe-CTCF + oe-NC group, the oe-H19 + oe-CTCF + oe-PKD1 group had a smaller area of atherosclerotic plaques, smooth inner walls of the artery, and more fibrous caps with no fracture. In addition, there was no distinct sign of fracture and hemorrhage within the atherosclerotic plaques, and there were more abundant smooth muscle cells and a larger content of type I and III collagen fiber. Moreover, a large number of foam cells accumulated in the atherosclerotic plaques. The cholesterol crystals were asymmetrically distributed, with calcification in some crystals. In general, the atherosclerotic plaques appeared to be in a stable state. The atherosclerotic plaques were less vulnerable, with a lower plaque vulnerability index (Figure 5C), suggesting that overexpressing PKD1 could partly salvage the vulnerable plaque formation caused by H19.

Immunohistochemical staining showed that CD34 was expressed in the vascular endothelial cells, suggesting its participation in intraplaque angiogenesis. A larger number of newly formed blood vessels were observed in the oe-H19 + oe-CTCF + oe-NC group compared to the oe-NC + oe-NC + oe-NC group (*p* < 0.05) (Figure 5D). Furthermore, Western blot analysis showed upregulated levels of the angiogenesis-related proteins MMP-2, VEGF and p53 and downregulated level of TIMP-1 in the oe-H19 + oe-CTCF + oe-NC group in comparison to the oe-NC + oe-NC+ oe-NC group (*p* < 0.05). There were no significant differences in the levels of angiogenesis-related proteins between the oe-H19 + oe-CTCF + oe-PKD1 group and the oe-NC + oe-NC + oe-NC group (Figure 5E). These results revealed the involvement of H19 in atherosclerotic vulnerable plaque formation and intraplaque angiogenesis through down-regulation of PKD1 upon recruitment of CTCF in ApoE knockout mice with AS.

## DISCUSSION

Despite efforts made in exploring pathogenesis and advancing therapeutic methods, AS still poses a great threat to human health around the world [20]. There is an evident association between intraplaque angiogenesis with destabilization of atherosclerotic plaques [21], whereby intraplaque angiogenesis has the potential to hinder internal atherosclerotic plaques from undergoing angiogenesis presenting an insight of vital importance for obtaining the stabilization for vulnerable plaques [22]. Elevated expression of H19 has been reported in human atherosclerotic plagues [23], and we aimed to investigate the functional role of H19, as well as its underlying regulatory mechanism in AS. Collectively, our results demonstrated that H19 promoted atherosclerotic vulnerable plaque formation and intraplaque angiogenesis by down-regulating PKD1 via recruitment of CTCF in an ApoE knockout mouse model of AS.

Initially, this study elucidated a high expression of H19 in aortic tissues of AS ApoE knockout mice, thereby suggesting the potential simulative role of H19 in pathogenesis of AS. Moreover, an upregulated plasma level of H19 has already been flagged as a potential risk factor for coronary artery disease in a Chinese population [24]. Another study has illustrated an association between common H19 polymorphisms with the risk and severity of coronary artery disease in a Chinese population, and high expression of H19 was observed in the patients with AS [25]. Our mouse AS aortic tissues illustrated inhibition of atherosclerotic vulnerable plaque formation following H19 silencing due to the maintenance of the atherosclerotic plaques in a stable state after silencing of H19. It has been emphasized that vulnerable plaques are pathologically characterized by big lipoprotein core (larger than 40% of plaques); thin fibrous cap; a higher proportion of inflammatory cells such as macrophages; less VSMCs; and a large number of newly formed blood vessels [26]. The formation and development of atherosclerotic vulnerable plaque are facilitated by the concurrent excruciating stress in the vessel wall [26]. Interestingly, a previous report reported that H19 silencing could hinder lipid accumulation and inflammation response in ox-LDL-treated macrophages in AS through up-regulation of miR-130b [27]. Additionally, an induced H19 knockdown impedes the ox-LDL stimulated proliferation and enhances the rate of apoptosis of human aorta VSMCs via modulation of the miR-148b/WNT/β-catenin axis [9]. These results provided a basis supporting our hypothesis that silencing of H19 could attenuate atherosclerotic vulnerable plaque formation in ApoE knockout mice.

In addition, we also demonstrated that AS aortic tissues with silencing of H19 had downregulated expression of the angiogenesis proteins MMP-2, VEGF and p53 and upregulated TIMP-1 expression. VEGF is highlighted as a predominant factor, with significance for AS development as well as a fundamental promoter of angiogenesis having a close relation with atherosclerotic plaque progression and vulnerability [28]. An aforementioned study has suggested that ghrelin inhibits intraplaque angiogenesis and enhances plaque stability through decreasing the expression of VEGF and VEGFR2 [21]. Underlined as one of the major proteinases in atherosclerotic plaque lesions, MMP-2 has been previously suggested to be a significant contributor for AS development in ApoE knockout mice [29]. TIMPs are endogenous protein regulators of the MMP family [30], promoting TIMP-2 expression contributes to retarded recruitment of monocytes to inflammatory sites including atherosclerotic plaques, interruption in intraplaque activity of MMP and facilitated stabilization of atherosclerotic plaque lesions [31]. Moreover, p53 has also been highlighted as a significant regulator of atherosclerotic plaque formation [32]. Knockdown of H19 has been revealed to inhibit glioma-induced angiogenesis and glioma-associated endothelial cell capabilities of proliferation, migration and tube formation by decreasing the expression of angiogenic factor vasohibin 2 through upregulation of miR-29a [33]. These results together suggested that H19 silencing might exercise an inhibitory effect on intraplaque angiogenesis through a reduction in the expression of MMP-2, VEGF and p53, with an increase in TIMP-1 expression in ApoE knockout mice.

**Figure 6:**
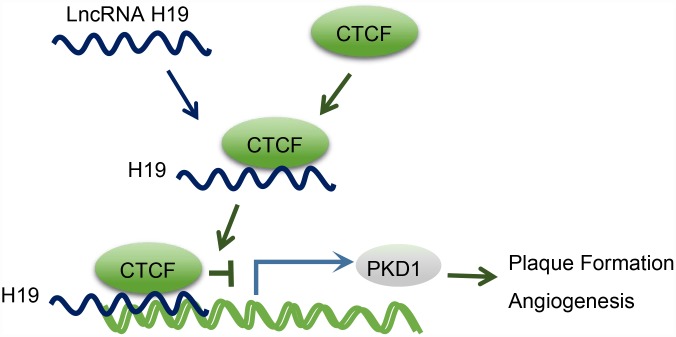
**The mechanism diagram illustrating that H19 participates in atherosclerotic vulnerable plaque formation and angiogenesis of mice with AS by inhibiting PKD1 expression via CTCF.**

Moreover, we found that PKD1 was downregulated in AS aortic tissues, which was reversed upon H19 silencing. Besides, CTCF could evidently bind to the promoter region of PKD1. Abnormal expression of PKD1 could thus potentially elevate the expression of active transcription factor [34]. CTCF is involved in the repression or activation of gene transcription with an array of DNA binding sites in the genome [35]. For instance, the involvement of CTCF in the HPV genome is of vital functionality in modulating the expression of early viral gene and transcript processing [36]. A previous study demonstrated that HOTTIP could regulate the expression of HOXA gene in accordance with CTCF [14]. Another lncRNA antisense non-coding RNA in the INK4 locus, which is central to AS formation, is also thought to exercise its trans-regulation functions by binding to CTCF [37]. Based on a genome-wide association study, PKD1 has been proposed as a novel gene for the prediction of coronary artery disease in patients with type 1 diabetes mellitus [17]. Besides, PKD1-deficient mice are susceptible to cardiac dysfunction, thus highlighting the significance of PKD1 in regard to cardiovascular disease [38]. A former study proposed that CTCF extensively combines the cis-acting enhancer regulatory regions and the promoter region of PKD2 by chromatin looping, thus enhancing PKD2 transcription [39], and conceivable effects of H19 on the expression of PKD2. Therefore, there are grounds for further seeking confirmation of the therapeutic potential of PKD1 for treating AS. Returning to our hypothesis proposed in the beginning of this study, we found that H19 was involved in atherosclerotic vulnerable plaque formation and intraplaque angiogenesis by down-regulating PKD1 through recruiting the CTCF transcription factor in ApoE knockout mice with AS (Figure 6). This finding elucidated the involvement of the H19/CTCF/PKD1 regulatory axis in atherosclerotic vulnerable plaque, hinting to the potential application of H19 in averting AS.

### Limitations

We found high expression of lncRNA H19 in mice with AS, while PKD1 was poorly expressed. Furthermore, the mechanistic analyses revealed that lncRNA H19 downregulated PKD1 expression via CTCF. Importantly, H19 promoted atherosclerotic vulnerable plaque formation and angiogenesis by down-regulating PKD1. All experiments were performed on mice, while the H19 expression in serum of patients with AS requires further verification for clinical application. Additionally, the effects of H19 on AS in transcriptome need to be examined by sequencing for an in-depth understanding. Existing literature has demonstrated a high expression of H19 in patients with cardiovascular disease [7, 25, 40, 41]. However, whether the underlying mechanism is in line with mice remains to be further studied.

### Future directions

We shall collect and analyze serum from patients with AS and healthy subjects, and perform transcriptome sequencing to retrieve the therapeutic target genes of AS. We shall design *in vitro* experiments on human cell lines to further unravel the function of the H19/CTCF/PKD1 axis in atherosclerotic vulnerable plaque formation and angiogenesis. In combination with AS-related genes obtained through transcriptome sequencing, the underlying mechanisms in AS will be explored, aiming to support the development of novel therapeutic agents of gene therapy.

## MATERIALS AND METHODS

### Ethic statement

The experimental procedures were conducted with approval of the Institutional Animal Care and Use Committee of Guizhou Provincial People’s Hospital. Mice were treated humanely, and adequate measures were taken to minimize their suffering.

### Establishment of AS model in ApoE knockout mice

A total of 132 ApoE knockout mice and 12 C57BL/6J mice (age: 6 - 8 weeks; weight: 20 - 30 g) acquired from the Guangdong Medical Laboratory Animal Center (Guangzhou, Guangdong, China) were chosen as study subjects. These mice were housed in separate cages for 24 weeks with free access to water and food (150 g food for each mouse every day). The 12 C57BL/6J mice were assigned as the control group with normal diet, and ApoE knockout mice were fed a high-fat diet to establish AS model. The high-fat food containing 21% fat and 0.15% cholesterol was processed from conventional food. Then, intermittent repeated smoking was instituted in ApoE knockout mice in order to increase atherosclerotic vulnerable plaque formation.

### Animal grouping

After successful establishment of AS models in ApoE knockout mice, the mice were grouped into the AS, negative control (NC)-antisense oligonucleotide (ASO), H19-ASO, oe-NC, oe-H19, oe-NC + sh-NC, oe-H19 + sh-NC, oe-H19 + sh-CTCF, oe-NC + oe-NC + oe-NC, oe-H19 + oe-CTCF + oe-NC, and oe-H19 + oe-CTCF + oe-PKD1 groups, with each group comprising of 12 mice. The following day after grouping, the mice were treated with drugs via an intraperitoneal injection for 13 weeks. The day after completion of the drug treatment regimen, the mice were anaesthetized by an intraperitoneal injection with 4 mL/kg 3% pentobarbital sodium. Next, the chest and the abdomen of mice were excised layer-by-layer using a scalpel and a pair of ophthalmic scissors under aseptic conditions. A section of the separated aorta was resected and fixed conventionally by immersion in 4% paraformaldehyde solution, dehydrated using gradient ethanol, cleared in xylene, and finally embedded with paraffin. A serial of sections of 5 μm thickness was sliced from the aortic root bottom, with 4 identical cross sections for each specimen. After baking at 60°C in an incubator for 6 h, the sections were kept at room temperature. The remaining section of the separated aorta was preserved in liquid nitrogen for further experimentation.

### RNA isolation and quantification

Trizol kit was used to extract the total RNA from the tissues, and the miRNeasy Mini Kit (217004, QIAGEN, Hilden, Germany) was used to extract the total RNA from the cells, respectively. Then, RNA was reversely transcribed into complementary DNA (cDNA) in strict accordance with the provided introductions of the PrimeScript RT kit (RR036A, Takara Biotechnology Ltd., Dalian, Liaoning, China). The cDNA was subjected to real time quantitative polymerase chain reaction (qPCR) according to the manufacture’s protocols provided by SYBR^®^ Premix Ex TaqTM II Kit (RR820A, Takara Biotechnology Ltd., Dalian, Liaoning, China) using an ABI7500 quantitative PCR instrument (7500, ABI Company, Oyster Bay, NY, USA). The primers for H19, CTCF, PKD1 and glyceraldehyde-3-phosphate dehydrogenase (GAPDH) were synthetized by Takara Biotechnology Ltd. (Dalian, Liaoning, China), as shown in Table 1. The 2^-ΔΔCt^ method was adopted to calculate the relative transcription levels of H19, CTCF, and PKD1 in respect of GAPDH as the internal reference.

**Table 1:** Primer sequences for RT-qPCR.

**Gene**	**Sequence (5′-3′)**
H19	F: GCAGGTAGAGCGAGTAGCTG
	R: CCTCTGCTGGAGACCCTAGT
CTCF	F: GTTGAAGCCATTGTGGAGGAGTCT
	R: ACCCCCATCTGTCTGGTTCTG
PKD1	F: TACTTCAGACGCTGGACATAGGG
	R: GGTTCCCACTCAGGTTTATTTCACT
GAPDH	F: CATGGCCTTCCGTGTTCCTA
	R: GCGGCACGTCAGATCCA

Note: LncRNA H19, long non-coding RNA H19; PKD1, polycystic kidney disease 1; CTCF, CCCTC-binding factor; GAPDH, glyceraldehyde-3-phosphate dehydrogenase; F, forward; R, reverse; RT-qPCR, reverse transcription quantitative polymerase chain reaction.

### Western blot analysis

The total protein in the tissues was extracted using the radio-immunoprecipitation assay lysis buffer (RIPA, R0010, Beijing Solarbio Science and Technology Co., Ltd., Beijing, China) containing phenylmethylsulfonyl fluoride (PMSF), followed by protein quantification. In total, 50 μg of protein was subjected to sodium dodecyl sulfate -polyacrylamide gel electrophoresis and transferred onto a polyvinylidene fluoride (PVDF) membrane. The membrane was blocked with 5% skimmed milk for 1 h and incubated at 4°C overnight with the following diluted primary antibodies against PKD1 (1 : 1000, bs-2157R), matrix metalloproteinase 2 (MMP-2) (1 : 1000, ab37150), vascular endothelial growth-factor (VEGF) (1 : 1000, ab46154), p53 (1 : 1000, ab131442), tissue inhibitor of metalloproteinases-1 (TIMP-1) (1 : 5000, ab38978), and GAPDH (1 : 5000, ab181602). Except for PKD1, which was purchased from Bioss Biotech (Beijing, China), the other antibodies were purchased from Abcam (Cambridge, MA, USA). After that, the membrane was incubated with the horseradish peroxidase-labeled goat anti-rabbit Immunoglobulin G (IgG) secondary antibody (1 : 1000, HA1003, Huiyan Biotech Co., Ltd., Shanghai, China) at room temperature for 1 h. Finally, membrane development was performed using enhanced chemiluminescence solution (ECL, 808-25, Biomiga, San Diego, CA, USA) for 1 min. The relative expression was expressed as the ratio of the gray value of the target band to that of the internal reference GAPDH band.

### Dual-luciferase reporter gene assay

The target gene of CTCF was analyzed and predicted by an online prediction website. The promoter region of PKD1 was introduced into the pGL3-Basic vector (Promega, Madison, WI, USA) to construct the pGL3-PKD1-wild-type (Wt). pGL3-PKD1-mutant (Mut) was also constructed with mutated potential binding sites of CTCF on the promoter of PKD1. The cells were inoculated in the 24-well plate at a density of 3 × 10^4^ cells/well and then transfected with pGL3-PKD1-Wt or pGL3-PKD1-Mut with oe-CTCF and CTCF-ASO as well as the Renilla plasmid. Finally, the luciferase activity was measured using the Dual-Luciferase Reporter Assay System (Promega, Madison, WI, USA) with the Renilla luciferase serving as an internal reference. The relative luciferase activity = Relative light unit (RLU) _firefly luciferase_ / RLU _Renilla luciferase_.

### Fluorescence in situ hybridization (FISH) assay

The subcellular localization of H19 in the aorta was identified by FISH assay in accordance with the instructions provided by Ribo^TM^ lncRNA FISH Probe Mix (Red) (RiboBio Co., Ltd., Guangzhou, Guangdong, China). The paraffin-embedded aorta sections were dewaxed using xylene A and xylene B (each for 10 min), immersed twice in absolute ethanol (5 min each), and detached using protease K at 55°C for 10 min. Subsequently, the sections were rinsed twice with 2 × saline sodium citrate (SSC) (5 min each), dehydrated at -20°C precooled alcohol (70%, 85%, and 100%; 5 min each) and then air-dried. The sections were denatured at 73°C for 5 min, dehydrated using -20°C precooled alcohol of variable concentrations (70%, 85%, and 100%; 5 min each) and air-dried. Then, the hybridization solution was added to the sections, denatured at 73°C for 5 min and further incubated at 37°C overnight. The following day, the sections were washed twice with 48°C preheated 50% formamide or 2 × SSC (5 min/time), rinsed twice using 2 × SSC (5 min/time), and counterstained with 4',6-diamidino-2-phenylindole staining solution for 5 min, followed by 2 × SSC washes (5 min/time). The sections were dried at room temperature in subdued light. Finally, 5 different fields were selected, observed and documented under a fluorescence microscope (Olympus, Tokyo, Japan).

### RNA binding protein immunoprecipitation (RIP) assay

The binding of H19 with CTCF was detected using the RIP kit (Millipore, Bedford, MA, USA). The tissues were rinsed using pre-cooled phosphate buffered saline (PBS) three times and homogenized. The tissues were then centrifuged at 1500 rpm for 5 min at 4°C and the supernatant was discarded, while the cells were acquired and further lysed using the RIPA lysis buffer (P0013B, Beyotime Biotechnology Co., Shanghai, China), immersed in an ice-bath for 5 min, and centrifuged at 14000 rpm for 10 min at 4°C for collection of the supernatant. A portion of the cell lysate was used as Input. Then, 50 μL of magnetic beads was rinsed and resuspended in 100 μL RIP wash buffer for each co-precipitation reaction system, and then incubated with 5 μg antibody against CTCF (5 μg/mg of lysate, ab70303) or IgG (1 : 100, ab172730) for co-precipitation. The magnetic bead-antibody complex was rinsed, resuspended in 900 μL RIP wash buffer and incubated with 100 μL cell extract at 4 °C overnight. The samples were placed on a magnetic base to collect the magnetic bead-protein complexes. Subsequently, the precipitated samples and inputs were detached using proteinase K to extract the RNA. H19 expression was detected by performing PCR. The antibodies utilized for RIP assay were CTCF (5 μg/mg of lysate, ab70303) and IgG (1 : 100, ab172730) as NC.

### Chromatin immunoprecipitation (ChIP) assay

The fresh frozen tissues were cut into 1 - 3 mm^2^ sections and cross-linked using 1% formaldehyde at room temperature for 15 - 20 min, followed by the addition of 2.5 M glycine to a final concentration of 0.125 M for termination of cross-linking. The sample was sonicated and diluted using 1.8 mL ChIP dilution buffer containing 1 mM PMSF to a final volume of 2 mL. A total of 20 μL sample was taken as Input. The remaining 2 mL sample was incubated with 70 μL of protein A + G agarose/salmon sperm DNA (about 35 μL of precipitate and 35 μL of liquid) at 4°C for 30 min. After centrifugation at 1000 × g for 1 min at 4°C, the supernatant was incubated with the CTCF antibody overnight. The following day, the sample was incubated with the magnetic beads, washed using washing solution and de-cross linked with 5 mmol/L NaCl. The DNA was recovered and detected using reverse transcription (RT)-qPCR with primers specific for PKD1 promoter.

### Hematoxylin-eosin (HE) staining

The paraffin-embedded sections were routinely dewaxed and hydrated. Then, the sections were dewaxed using xylene I and II (each for 10 min), and rehydrated in gradient ethanol (100%, 95%, 80%, and 70%, 2 min for each concentration),. After hydration, the sections were conventionally stained with HE (Beijing Solarbio Science and Technology Co., Ltd., Beijing, China), then dehydrated using gradient alcohol, cleared using xylene, and sealed with neutral gum. The aortic plaque formation in mice aortic root was observed under an optical microscope (XP-330, Shanghai Bingyu Optical Instrument Co., Ltd., Shanghai, China).

Evaluation of plaque stability: the pathological sections of mice were processed under HE staining, and the extracellular lipid components (mainly cholesterol crystals and cholesteryl ester) within the aortic plaques of mice in each group were observed under an optical microscope. The sections were further processed with modified movat pentachrome staining, and the smooth muscle cells, foam cells, collagen components and fibrous cap burial in the atherosclerotic plaque of each group were observed under a light microscope. Using of the Medical Image Analysis System, the ratio of extracellular lipids, foam cells, collagen components, and smooth muscle cells to the area of atherosclerotic plaque was calculated according to the following formula: plaque vulnerability index = (extracellular lipid components + foam cells) / (smooth muscle cells + collagen fibers). Observation of the histological pictures was performed in a double-blind manner.

### Detection of microvessel density (MVD)

The paraffin-embedded sections were routinely dewaxed and hydrated. Then, the sections were dewaxed with xylene I and II (each for 10 min) and hydrated in gradient ethanol (100%, 95%, 80%, and 70%, 2 min/concentration). Next, the sections were immersed in 3% H_2_O_2_ for 10 min, treated with high-pressure antigen retrieval for 90 s, and cooled at room temperature. After that, the sections were blocked with 5% bovine serum albumin solution for 30 min at 37°C, followed by overnight incubation at 4 °C with 50 μL of the polyclonal antibody rabbit anti-mouse CD34 (1 : 4000, ab81289, Abcam, Cambridge, MA, USA) or PBS as NC. Subsequently, the sections were further incubated with 50 μL biotinylated goat anti-rabbit IgG (1 : 100, ab172730, Abcam, Cambridge, MA, USA) for 30 min at 37°C. Next, the sections were colored using diaminobenzidine and counterstained with hematoxylin for 5 min, followed by microscopic observation. The criteria used to assess the results of immunohistochemical staining was as follows [42]: the positive cells were reflected as those with a staining degree of higher than 25% and the predominant localization of the staining area in the cytoplasm or cell membrane and vascular endothelium, which was brownish yellow in color. Counting of MVD was based on the number of CD34 positive cells in the field of view. The tan or brownish yellow stained endothelial cell cluster or a single endothelial cell, with a clear demarcation from the adjacent cells, microvessels, and surrounding connective tissue, was counted as a microvessel. If no connections between the structures were evident, their branched anatomical structure could be regarded as a blood vessel. With five high randomly magnification fields selected, the positive expression of CD34 and MVD was determined. Observation of the histological pictures was performed in a double-blind manner.

### Statistical analysis

Statistical analysis was conducted using the SPSS 21.0 software (IBM Corp, Armonk, N.Y., USA). Measurement data were presented as mean ± standard deviation, and the enumeration data were expressed as percentage or ratio and tested by χ^2^. The normal distribution of data was tested by the Kolmogorov-Smirnov method. If the data followed normal distribution, comparisons between multiple groups were analyzed by one-way analysis of variance (ANOVA), followed by the Tukey post hoc test. If the data showed a skew distribution, comparisons between multiple groups were analyzed by the Kruskal-Wallis test, followed by Dunn's post hoc test. A value of *p* < 0.05 was statistically significant.

## Supplementary Material

Supplementary Figures
