# Correction for: Silencing of long non-coding RNA H19 downregulates CTCF to protect against atherosclerosis by upregulating PKD1 expression in ApoE knockout mice

**DOI:** 10.18632/aging.203391

**Published:** 2021-07-31

**Authors:** Yongyao Yang, Feng Tang, Fang Wei, Long Yang, Chunyan Kuang, Hongming Zhang, Jiusheng Deng, Qiang Wu

**Affiliations:** 1 Department of Cardiology, Guizhou Provincial People’s Hospital, Guiyang, 550002, P. R. China; 2 Department of Cardiology, The General Hospital of Ji’nan Military Region, Ji’nan, 250031, P. R. China; 3 Department of Pathology and Laboratory Medicine, Emory University School of Medicine, Atlanta, GA 30322, USA

**Keywords:** correction

Original article: Aging. 2019; 11:10016–10030.  . https://doi.org/10.18632/aging.102388

**This article has been corrected:** The authors replaced the “oe-H19 + oe-CTCF + oe-PKD1” panel of the HE staining in **Figure 5C**, which was accidently mislabeled and partially duplicated with the “oe-NC + oe-NC + oe-NC” image. Replacement was done using representative images from the original sets of experiments. This alteration does not affect the results or conclusions of this work.

The new **Figure 5** is presented below.

**Figure 5:**
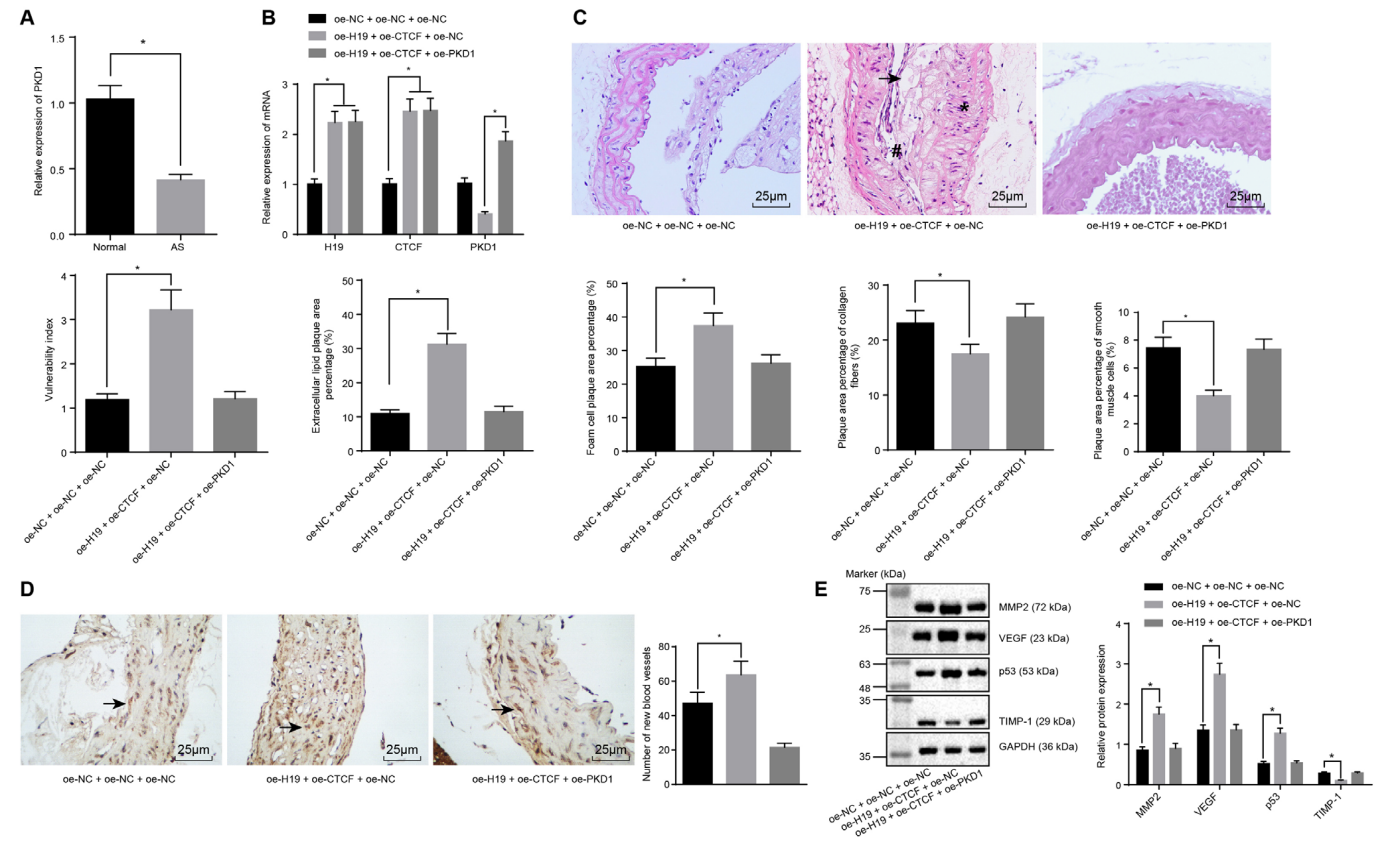
**H19 is involved in atherosclerotic vulnerable plaque formation and intraplaque angiogenesis through down-regulating PKD1 by recruiting CTCF in ApoE knockout mice with AS.** (**A**) The expression pattern of PKD1 in the aortic tissues of normal and AS mice determined by RT-qPCR. * *p* < 0.05 *vs.* the control group. (**B**) The overexpressing efficiency of H19, CTCF and PKD1 assessed by RT-qPCR. * *p* < 0.05 *vs.* the oe-NC + oe-NC + oe-NC group; # *p* < 0.05 *vs.* the oe-H19 + oe-CTCF + oe-NC group. (**C**) The atherosclerotic vulnerable plaque formation evaluated by HE staining (× 400) (The arrow referred to lipid vacuoles, * represented inflammatory cells and # indicated fractured smooth muscle). (**D**) The number of new blood vessels measured by Immunohistochemical staining (× 400) (The arrow referred to CD34-positive cells). (**E**) The protein levels of MMP-2, VEGF, p53 and TIMP-1 in atherosclerotic plaques normalized to GAPDH after transfection determined by Western blot analysis. * *p* < 0.05 *vs.* the oe-NC + oe-NC + oe-NC group. The data were measurement data and expressed by mean ± standard deviation. Data differences between two groups were analyzed by unpaired *t*-test; comparisons made among multiple groups were analyzed by one-way ANOVA. The experiments were repeated three times independently.

