# Supplementary material for: Silencing of long non-coding RNA H19 downregulates CTCF to protect against atherosclerosis by upregulating PKD1 expression in ApoE knockout mice
Source: Aging (Albany NY). 2019 Nov 22;11(22):10016–30. doi: 10.18632/aging.102388 (PMC6914395; doi:10.18632/aging.102388)
Supplement: Supplementary Figures [file aging-11-102388-s001..pdf]

SUPPLEMENTARY FIGURES

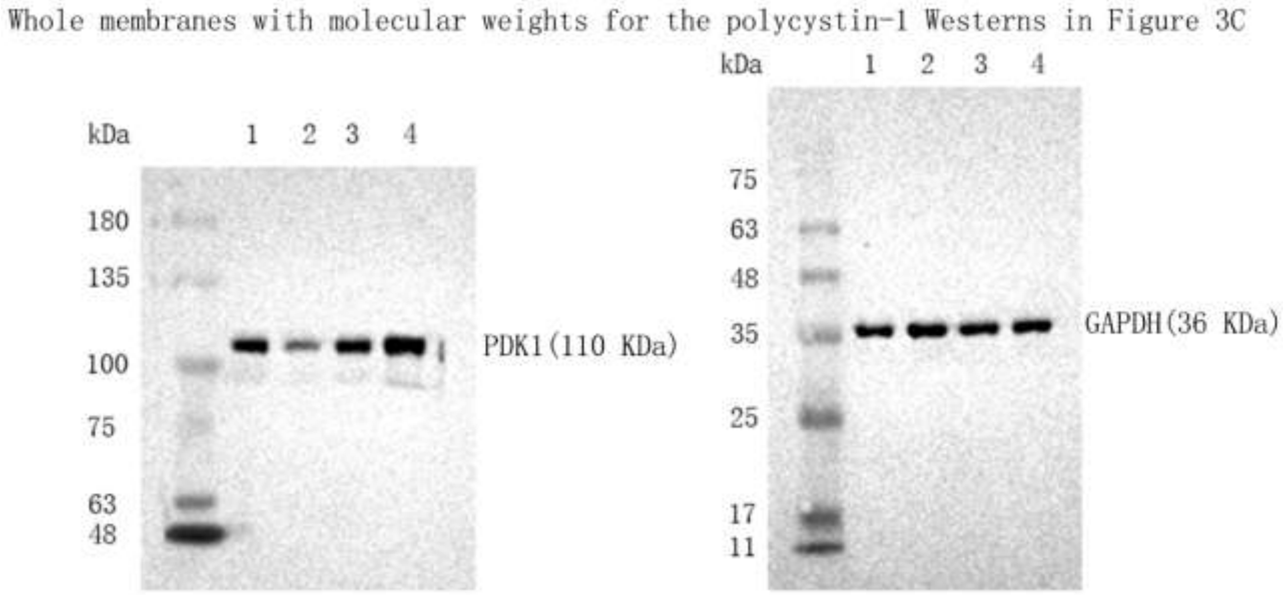

Supplementary Figure 1. Whole membranes with molecular weights for the polycystin-1 westerns in Figure 3C.

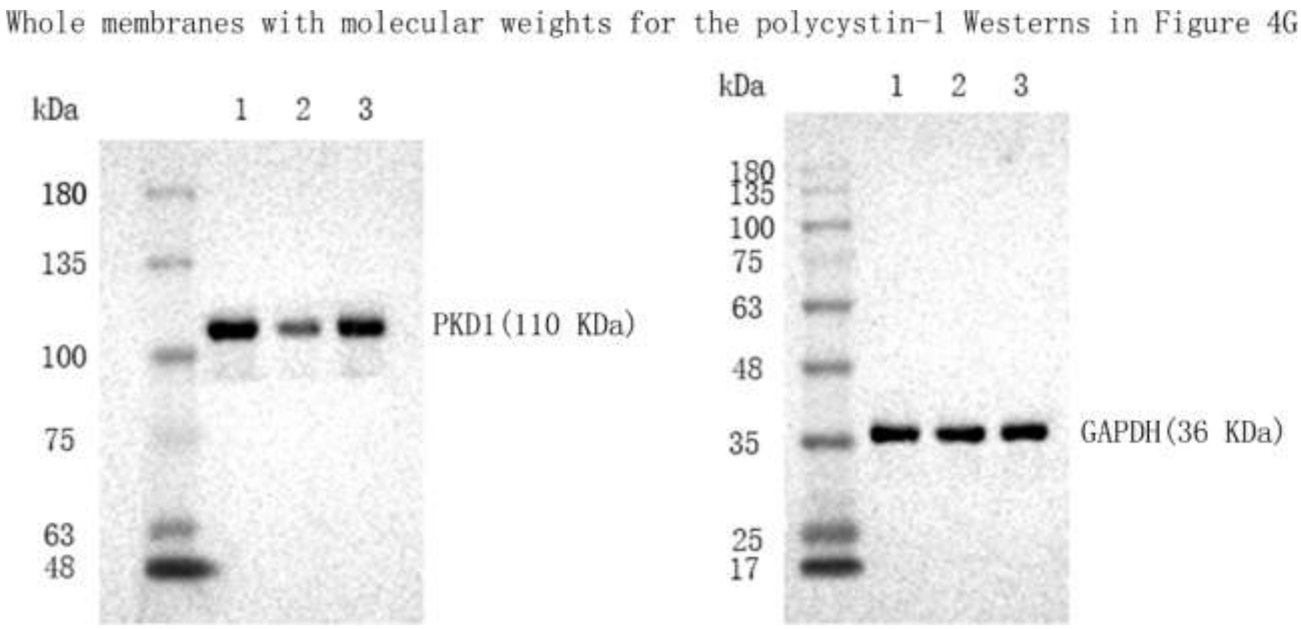

Supplementary Figure 2. Whole membranes with molecular weights for the polycystin-1 westerns in Figure 4G.
